# Supplementary material for: Dynamic character displacement among a pair of bacterial phyllosphere commensals in situ
Source: Nat Commun. 2022 May 20;13:2836. doi: 10.1038/s41467-022-30469-3 (PMC9123166; doi:10.1038/s41467-022-30469-3)
Supplement: Supplementary file 1 — Supplementary Information [file 41467_2022_30469_MOESM1_ESM.pdf]

Dynamic character displacement among a pair of bacterial phyllosphere commensals *in situ*

Hemmerle *et al.*

Supplementary Information

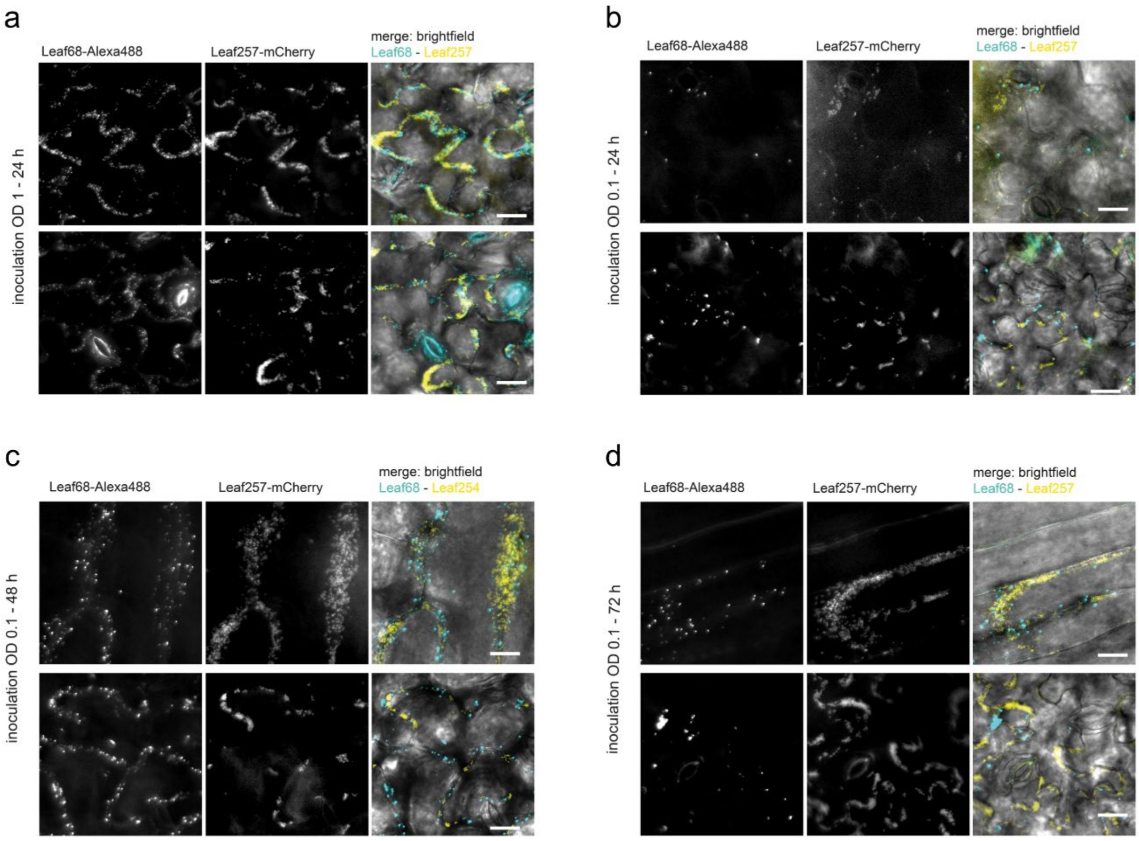

Supplementary Fig. 1: Microscopy images of *Rhizobium* Leaf68 and *Sphingomonas* Leaf257 during co-colonization of the Arabidopsis phyllosphere. For visualization, *Rhizobium* Leaf68 was stained with an Alexa F488 dye and *Sphingomonas* Leaf257 constitutively produced mCherry (see Material and Methods). a) 24 hours after inoculation with OD_{600}=1 showing that co-localization is obtained by the inoculation method (see Material and Methods). Second experiment using OD_{600}=0.1 and imaged after b) 24 hours, c) 48 hours, d) 72 hours showing that the co-occurrence is stable over time. Notably, the Alexa F488-dye is reduced to the limit of detection after 72 hours. For a-d the scale bar (white line) is 20 µm. Representative images from two leaves from two different plants (upper and lower panel) from a single plant experiment are shown.

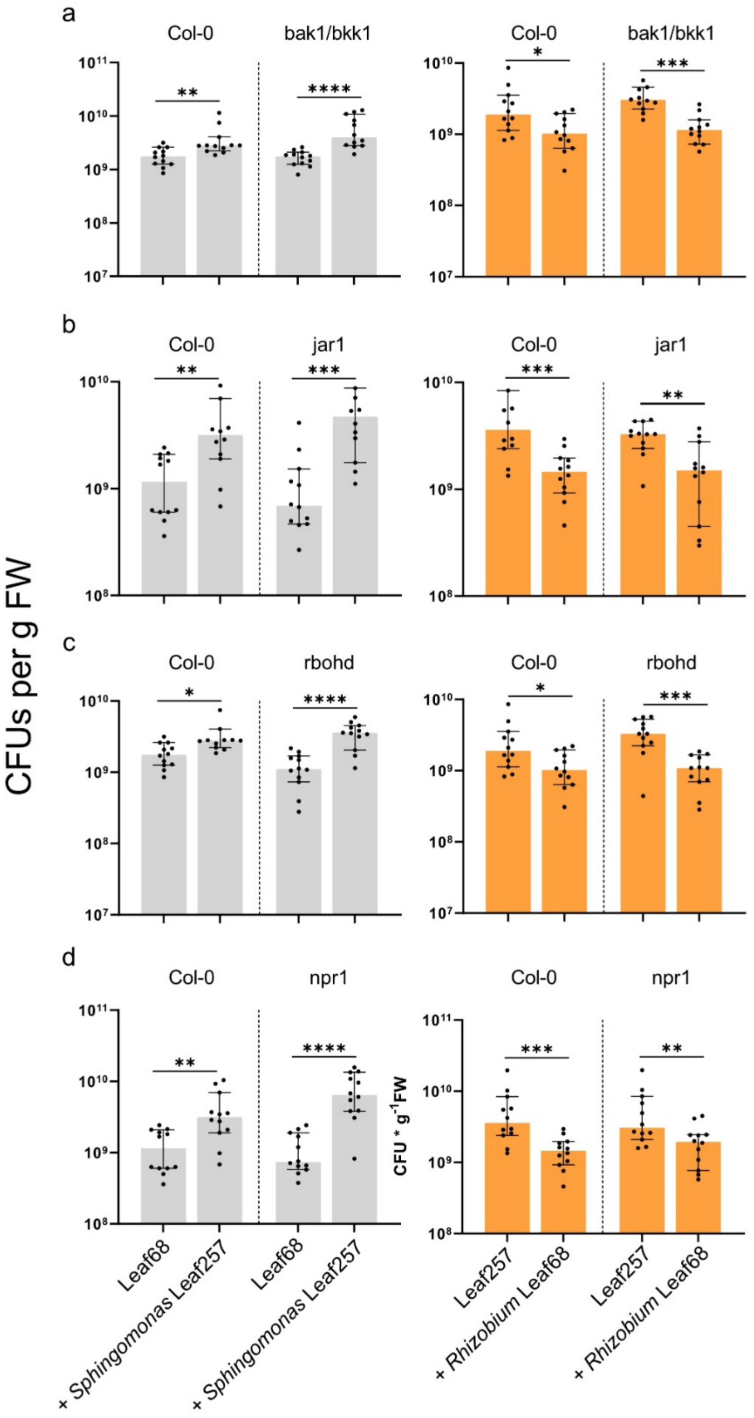

Supplementary Fig. 2: Interaction of Leaf68 and Leaf257 in the phyllosphere over different plant immunity mutants. Graphs depict the cell numbers (CFUs per gram fresh weight) of *Rhizobium* Leaf68 (gray) and *Sphingomonas* Leaf257 (orange) upon mono- and co-colonization in Col-0 and different plant immunity mutants: a) the co-receptor mutant *bak1/bkk1* involved in the pattern-triggered immunity, b) the plant mutant *jar1* involved in the jasmonic-acid (JA signaling), c) the respiratory burst oxidase mutant *rbohd* involved in the ROS-dependent plant defense and d) the immunity mutant *npr1* involved in the onset of systemic acquired resistance (SAR). For all graphs, each dot represents CFU from a single plant with twelve plants in total (n=12). The median and 95% confidence interval are shown. Statistical analysis was performed using Kurskal-Wallis test (p-values: * p-value < 0.05, ** p-value < 0.01, *** p-value < 0.001; **** p-value < 0.0001). Source data are provided as a Source data file.

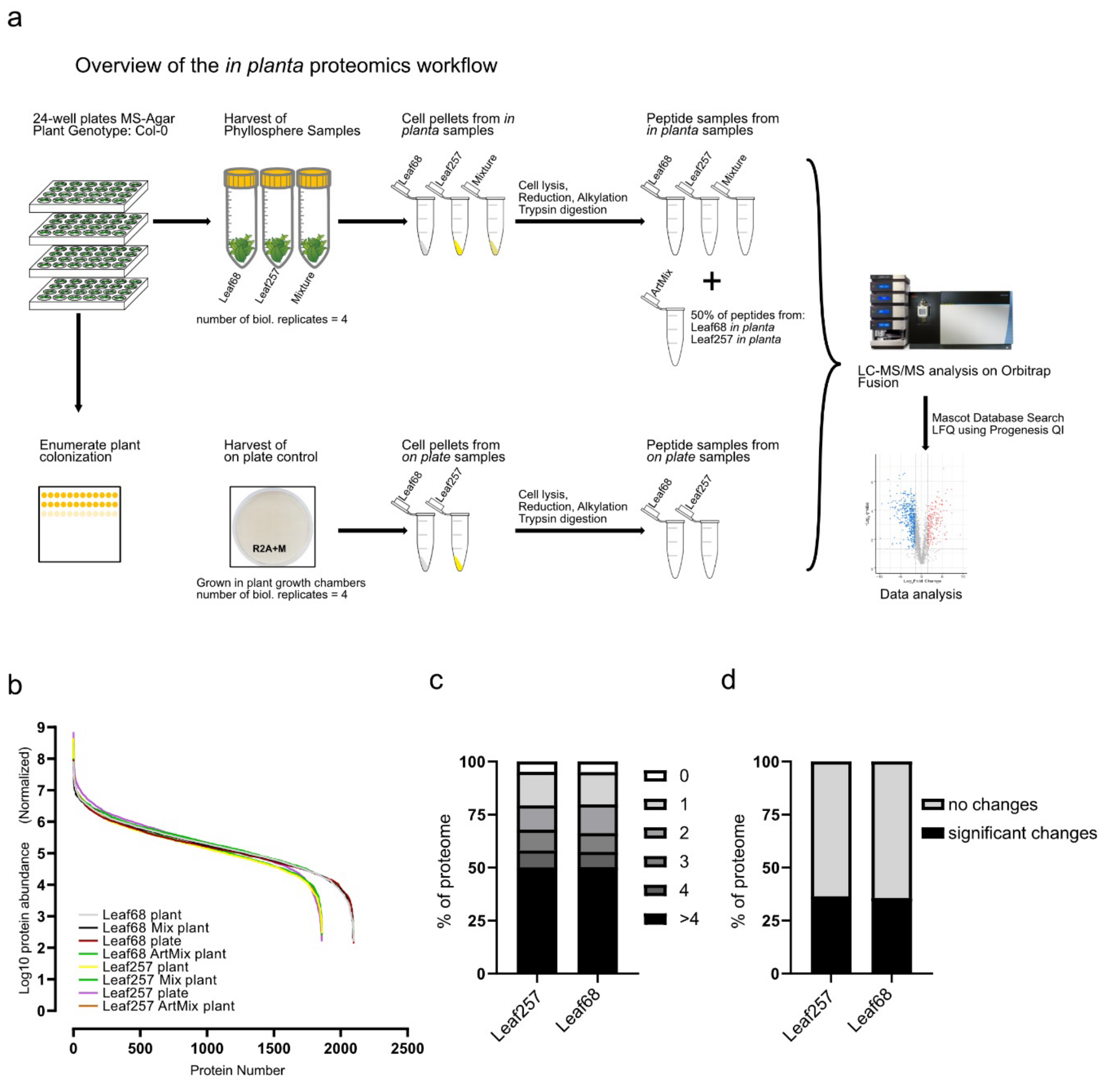

Supplementary Fig. 3: Proteomics workflow and overview of the label-free quantification (LFQ). a) Schematic overview of experimental workflow to determine the proteome of the two phyllosphere commensals *Sphingomonas* Leaf257 and *Rhizobium* Leaf68 alone and during co-colonization of the plant phyllosphere. b) Normalized protein abundance over all samples. Protein abundance was estimated using a Hi-3 approach (see methods). The mixture is abbreviated as Mix and the artificial mixture as ArtMix. c) Fraction of proteins identified with 0, 1, 2, 3 and more than 4 unique peptides per protein. 100% corresponds to all measured proteins. d). Fraction of quantified and regulated proteins. 100% corresponds to all proteins identified/quantified with at least two unique peptides. Source data for (b,c,d) are provided as a Source Data file.

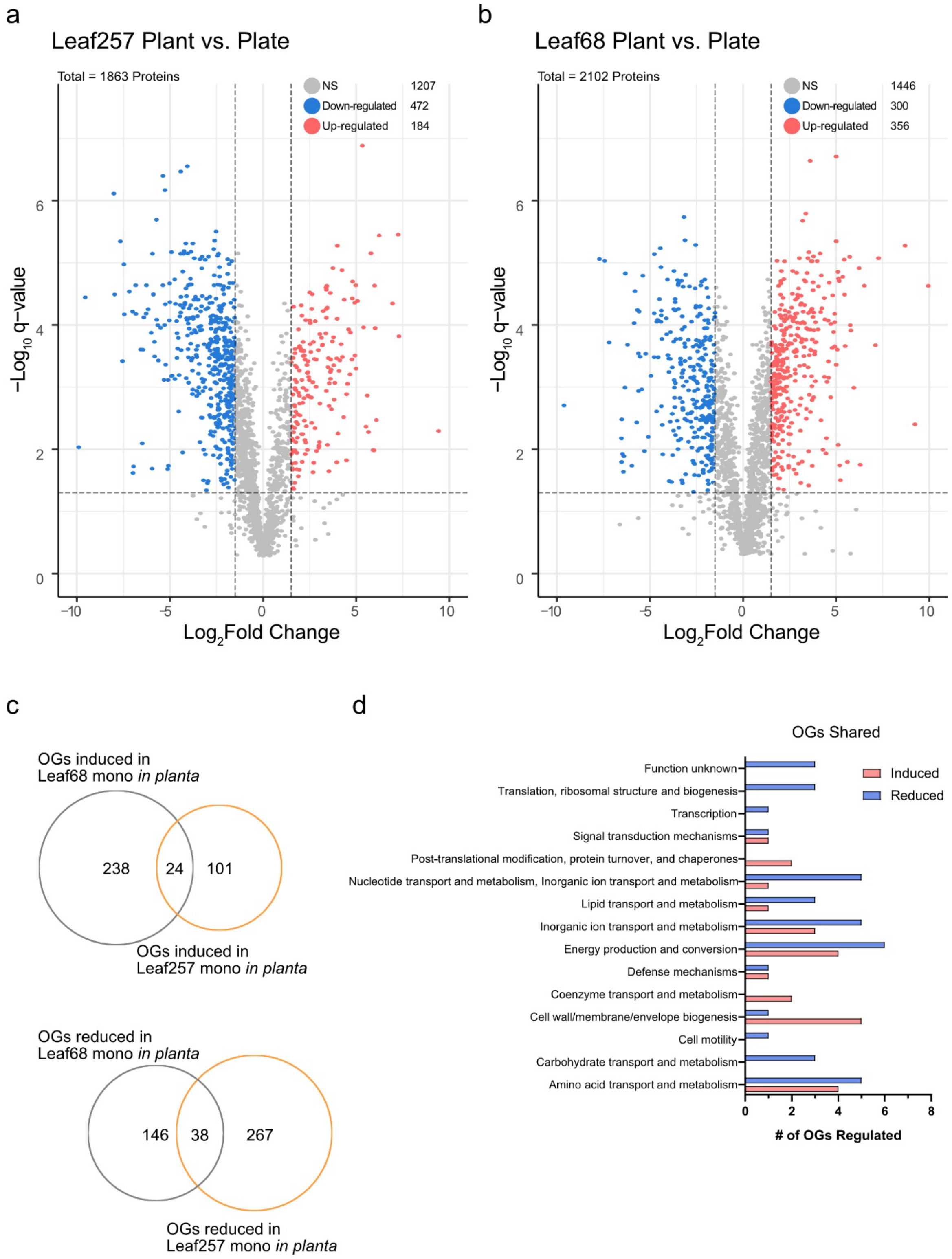

Supplementary Fig. 4*: Proteome changes during colonization of the plant alone compared to on plate control*. a) Volcano plot of *Sphingomonas* Leaf257 during mono-colonization of the plant compared to plate control. b) Volcano plot of *Rhizobium* Leaf68 during mono-colonization of the plant compared to plate control. For both volcano plots the data depicts four independent biological replicates

(n=4). The adjusted p-value (see methods) (q-value) cutoff was set to 0.05 (horizontal line) and Log_2 Fold change cutoffs are set to > 1.5 or < -1.5 (vertical lines). In the graphs, only proteins detected with at least two unique peptides are shown. The total amount of proteins, not significantly regulated (NS), and Down- / Up-regulated are indicated in each graph. For a) and b) statistical analysis was performed using one-way ANOVA and resulting *P*-values were corrected for multiple comparisons using Benjamini-Hochberg. c) Number of orthologous groups (OGs) significantly induced (top) and reduced (bottom) in Leaf68 and Leaf257. The overlap represents the OGs shared between each other, i.e. 24 OGs (top) and 38 OGs (bottom). d) Bar plot depicting the functional annotation of shared OGs significantly induced/reduced. For the comparison, only proteins detected with at least three unique peptides and with a Log_2 fold-change > 1.5 or < -1.5, q-value < 0.05 were considered. Source data are provided as a Source data file.

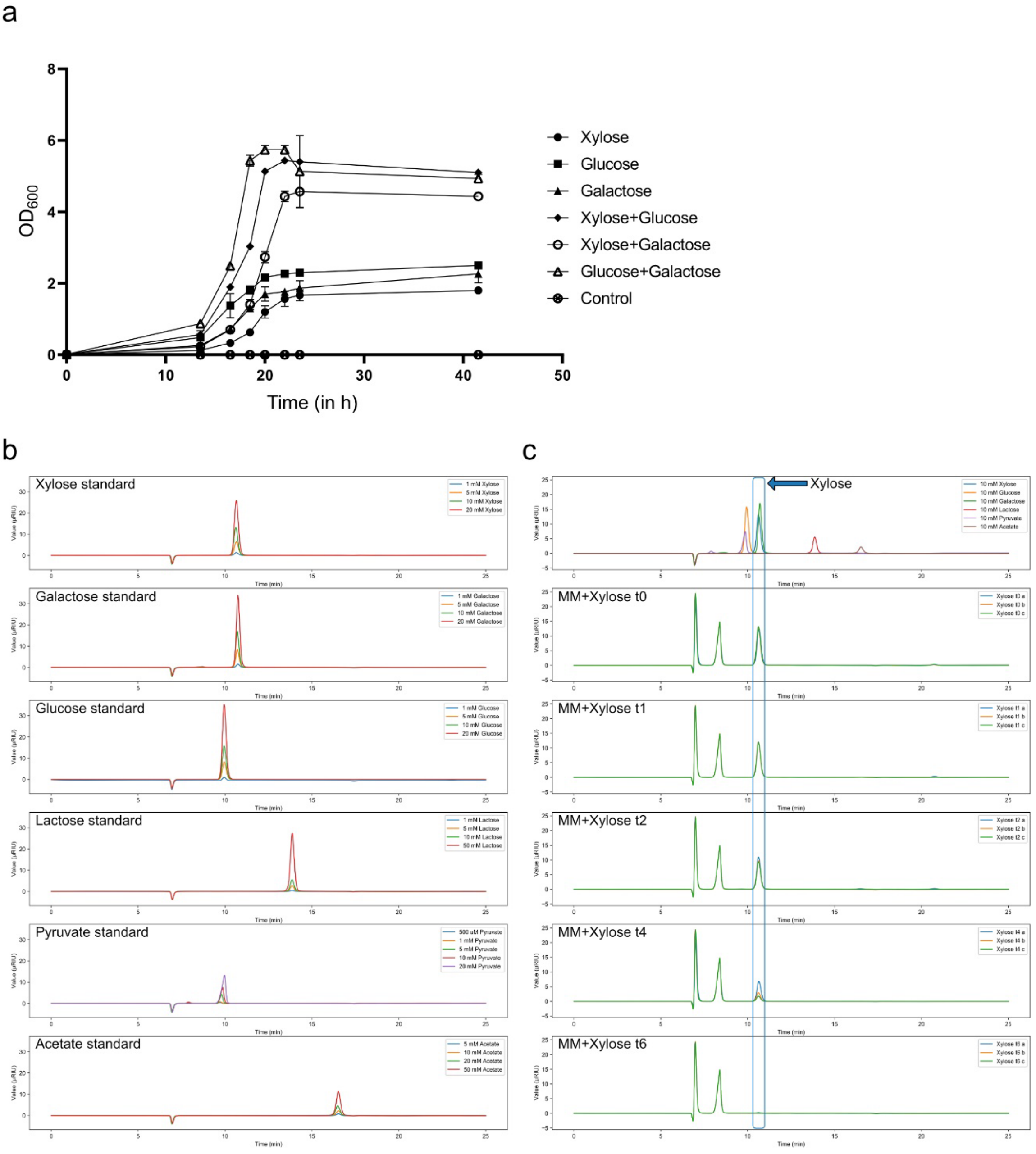

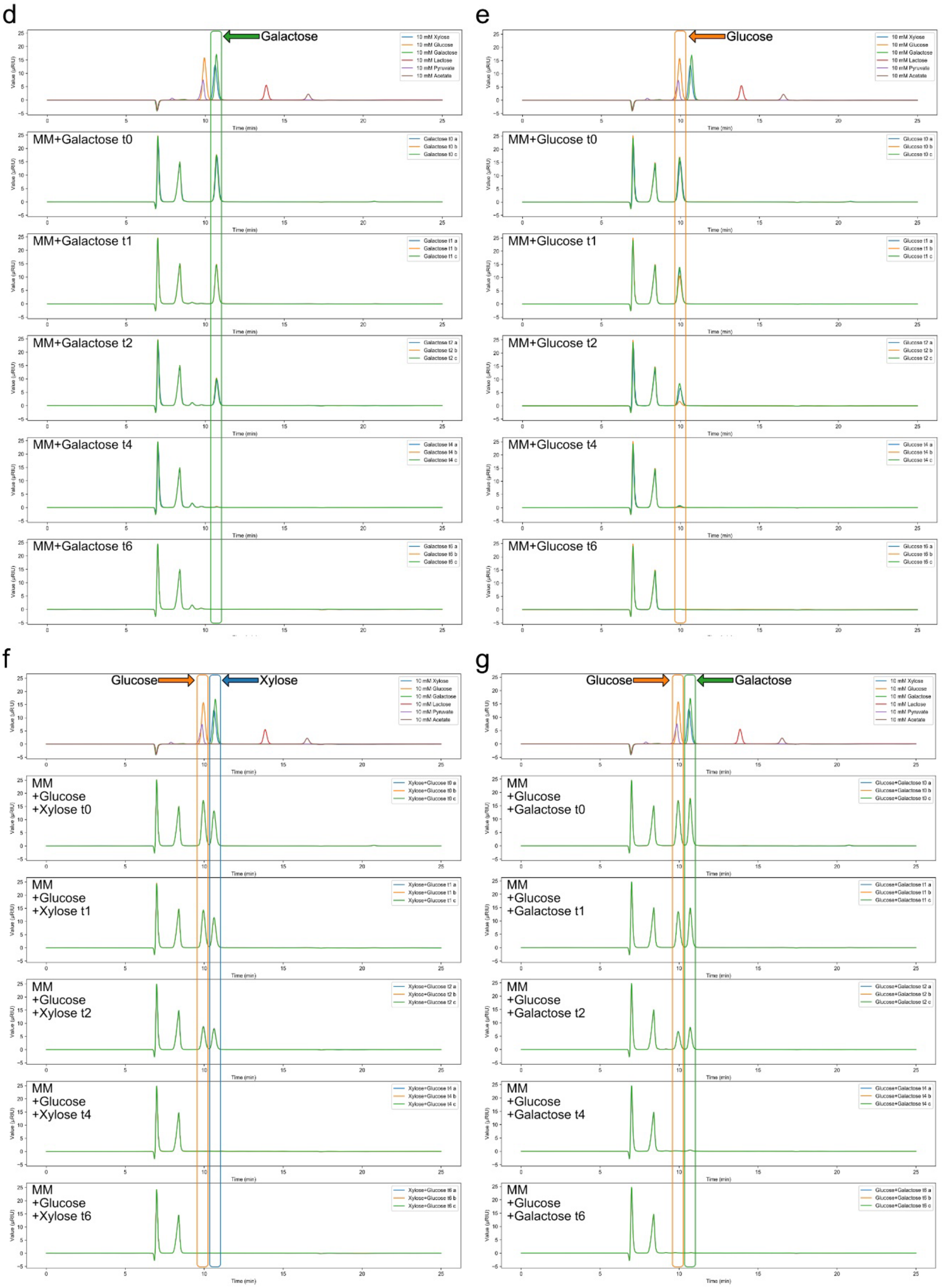

Supplementary Fig. 5: Supernatant analysis of *Sphingomonas* Leaf257 to determine its sugar preferences. a) (n = 3) Growth experiment and origin of supernatant samples for HPLC. *Sphingomonas* Leaf257 was cultivated in minimal medium containing xylose, glucose, galactose or binary combinations with each sugar at a concentration of 10 mM. Therefore, the OD_{600} in sugar combinations is twice as high (two sugars at 10 mM overall C-source abundance is 20 mM). Error bars represent the standard deviation. b)-g) Supernatant analysis by HPLC (see methods). Source data are provided as a Source data file.

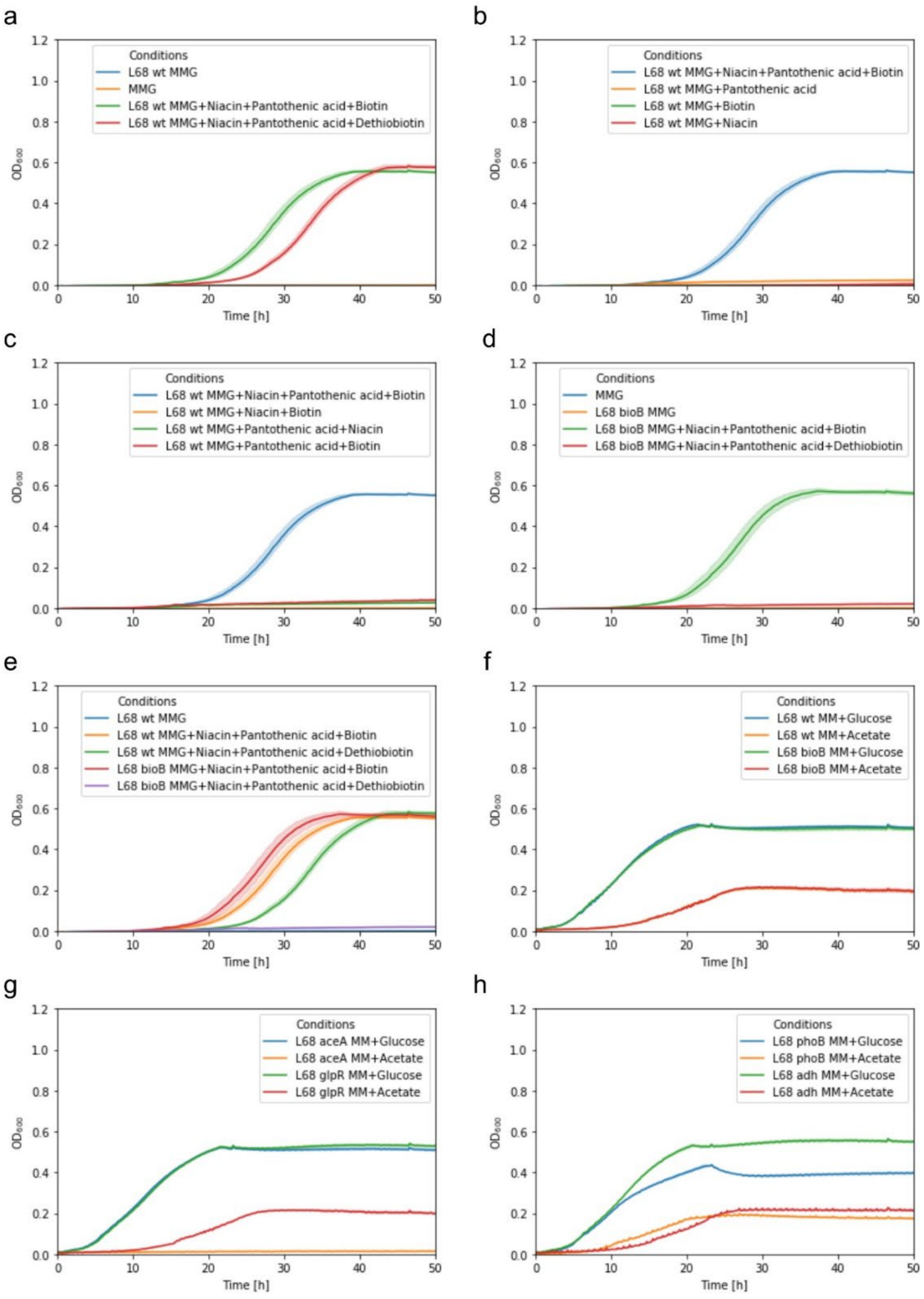

Supplementary Fig. 6: a) Growth curves of Leaf68 wild type on minimal medium with glucose (MMG) without and with addition of the three vitamins as well as the biotion precursor dethiobiotin. b) Depiction of Leaf68 wild type testing growth on each vitamin supplemented alone. c) Binary Combination of vitamis does not supplement growth. Only adding all three at the same time leads to growth. d) Leaf68 *bioB::Km*^{R} cannot grow with dethiobiotin but still on biotin, confirming the knock out of the biotin synthase. e) Leaf68 wild type and Leaf68 *bioB::Km*^{R} plotted together. f) Leaf68 wild type and Leaf68 *bioB::Km*^{R} in MM+Glucose and MM+Acetate. g) Leaf68 *aceA::Km*^{F} and Leaf68 *glpR::Km*^{F} in MM+Glucose and MM+Glycerol. h) Leaf68 *phoB::Km*^{F} and Leaf68 *adh::Km*^{F} in MM+Glucose and MM+Acetat. Vitamin auxotrophy data of *Rhizobium* Leaf68 were obtained from personal communication (Lehtinen unpublished). For all graphs five biological replicates (n = 5) were obtained. In all graphs the error bars represent the standard deviation. Source data are provided as a Source data file.

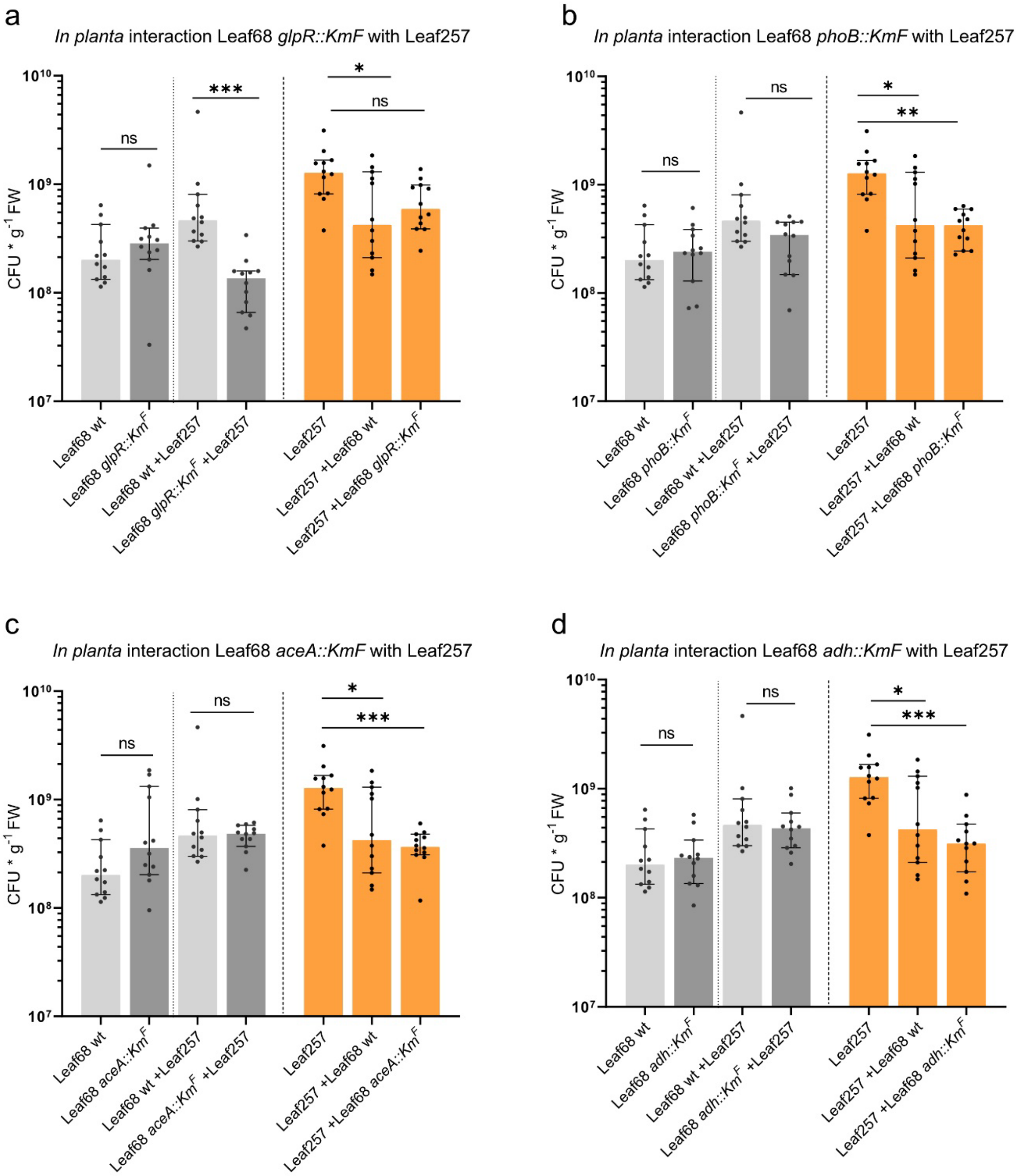

Supplementary Fig. 7: Colonization overview of *Rhizobium* Leaf68 mutants and their influence on *Sphingomonas* Leaf257. Graphs depict the cell counts (CFUs per gram fresh weight) for Leaf68 wild type (light gray), each Leaf68 mutant tested (dark gray) and the combinations with *Sphingomonas* Leaf257 (orange) *in planta*. Data points were obtained from one plant experiment with each data point representing a single plant (n=12). For each graph, the median and 95% confidence interval are depicted. Statistical analysis was performed using Kruskal-Wallis (p-values: * p-value < 0.05, ** p-value < 0.01, *** p-value < 0.001). a) Overview of Leaf68 *glpR::Km^F* vs Leaf257. b) Leaf68 *phoB::Km^F* vs Leaf257. c) Leaf68 *aceA::Km^F* vs Leaf257 and d) Leaf68 *adh::Km^F* vs. Leaf257. Source data are provided as a Source data file.

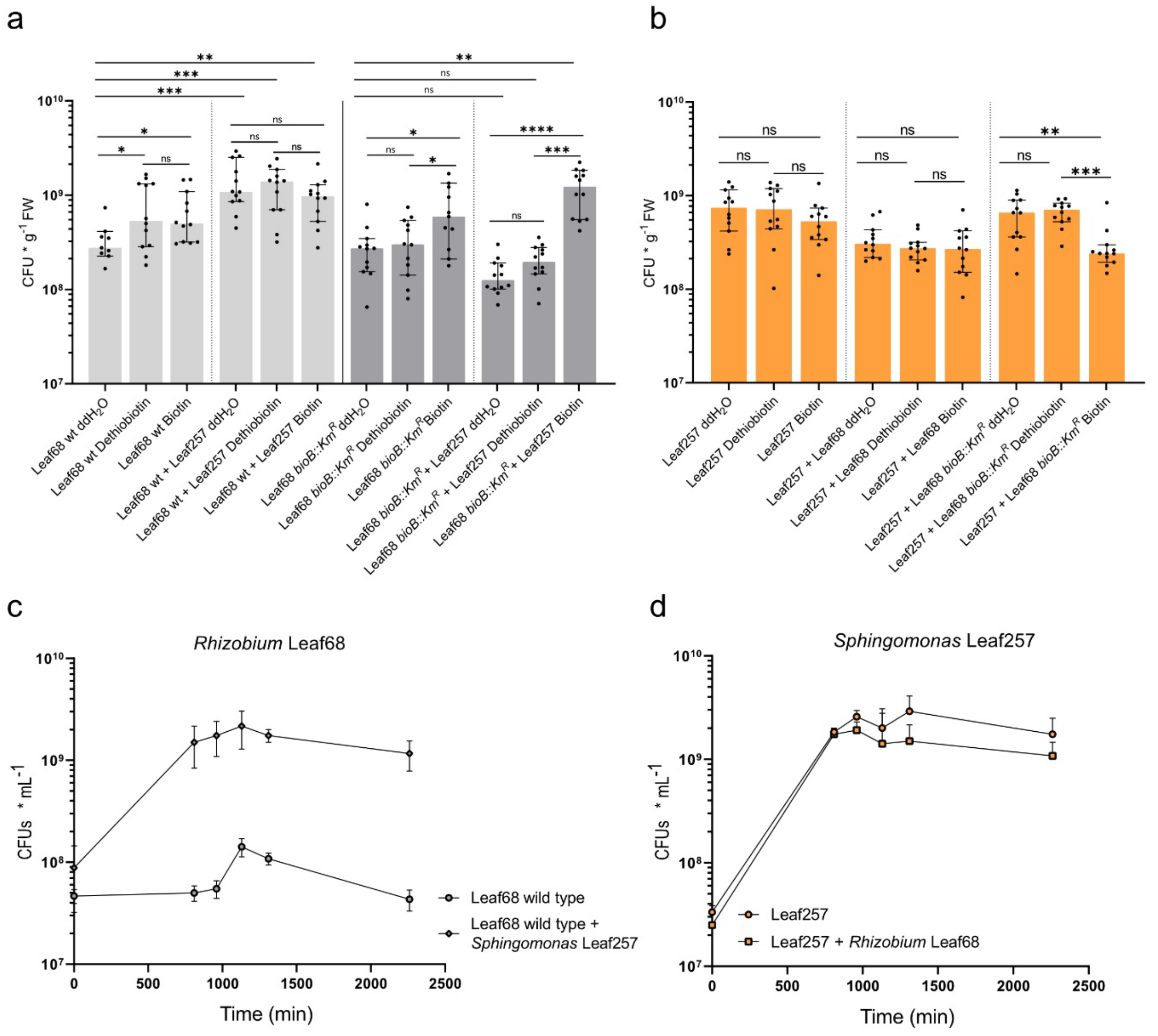

Supplementary Fig. 8: Colonization capacity (CFUs per gram fresh weight) obtained from the different treatments (ddH$_2$O, dethiobiotin and biotin) during mono- and co-colonization of a) *Rhizobium* Leaf68 wild type (light gray) and *Rhizobium* Leaf68 *bioB::Km^R* (dark gray) and b) *Sphingomonas* Leaf257. Each data point represents the CFUs of a single plant with 12 data points in total. In the graph, the median and 95% confidence interval are shown Statistical analysis was performed using the Kruskal-Wallis-test (p-values: * p-value < 0.05, ** p-value < 0.01, *** p-value < 0.001). Cell numbers (CFUs per mL) from shake flasks containing minimal medium and 10 mM glucose for c) *Rhizobium* Leaf68 wild type grown alone, and during co-cultivation with Leaf257. d) *Sphingomonas* Leaf257 grown alone, and during co-cultivation with *Rhizobium* Leaf68 wild type. Data represents three independent biological replicates (n=3). For each data point the mean and standard deviation (SD) are shown. Source data are provided as a Source data file.
