# Supplementary material for: Dynamic character displacement among a pair of bacterial phyllosphere commensals in situ
Source: Nat Commun. 2022 May 20;13:2836. doi: 10.1038/s41467-022-30469-3 (PMC9123166; doi:10.1038/s41467-022-30469-3)
Supplement: Supplementary file 3 — Description of Additional Supplementary Information [file 41467_2022_30469_MOESM3_ESM.pdf]

Supplementary Data 1: List of proteins quantified during colonization of the phyllosphere (*in planta*) compared to artificial media for *Sphingomonas* Leaf257. Only proteins detected with at least two unique peptides were considered for quantification.
Sheet 1: List of proteins for *Sphingomonas* Leaf257 considered significantly changing with the cutoffs q-value of < 0.05 and a Log_2 fold-change ≥ 1.5 or ≤ -1.5.
Sheet 2: List of all proteins identified with two or more unique peptides in *Sphingomonas* Leaf257.

Supplementary Data 2: List of proteins quantified during colonization of the phyllosphere (*in planta*) compared to artificial media for *Rhizobium* Leaf68. Only proteins detected with at least two unique peptides were considered for quantification.
Sheet 1: List of proteins for *Rhizobium* Leaf68 considered significantly changing with the cutoffs q-value of < 0.05 and a Log_2 fold-change ≥ 1.5 or ≤ -1.5.
Sheet 2: List of all proteins identified with two or more unique peptides in *Rhizobium* Leaf68.

Supplementary Data 3: List of COG-terms shared between *Rhizobium* Leaf68 and *Sphingomonas* Leaf257 and significantly changing *in planta* compared to artificial media. A Log_2 fold-change ≥ 1.5 or ≤ -1.5 was used as abundance change cutoff.
Sheet 1: Shared COG-terms induced *in planta* compared to artificial media.
Sheet 2: Shared COG-terms reduced *in planta* compared to artificial media.

Supplementary Data 4: List of proteins quantified during co-colonization compared to mono-colonization (artificial mixture) of the phyllosphere for *Sphingomonas* Leaf257. Only proteins detected with at least two unique peptides were considered for quantification.
Sheet 1: List of proteins for *Sphingomonas* Leaf257 considered significantly changing with the cutoffs q-value of < 0.05 and a Log_2 fold-change ≥ 1.5 or ≤ -1.5.
Sheet 2: List of all proteins identified with two or more unique peptides in *Rhizobium* Leaf68. Sheet 3: List of proteins detected for the Shikimate pathway.

Supplementary Data 5: List of proteins quantified during co-colonization compared to mono-colonization (artificial mixture) of the phyllosphere for *Rhizobium* Leaf68. Only proteins detected with at least two unique peptides were considered for quantification.
Sheet 1: List of proteins for *Rhizobium* Leaf68 considered significantly changing with the cutoffs q-value of < 0.05 and a Log_2 fold-change ≥ 1.5 or ≤ -1.5.
Sheet 2: List of all proteins identified with two or more unique peptides in *Rhizobium* Leaf68.

Supplementary Data 6: List of primers used in this work.
